# Retraction Note: Histone methyltransferase SETD1A interacts with notch and promotes notch transactivation to augment ovarian cancer development

**DOI:** 10.1186/s12885-026-16734-4

**Published:** 2026-08-14

**Authors:** Hongjuan Chai, Chunpeng Pan, Mingyang Zhang, Haizhong Huo, Haiyan Shan, Jugang Wu

**Affiliations:** 1 Department of Gynecology and Obstetrics, Shanghai Ninth People’s Hospital, Shanghai JiaoTong University School of Medicine, Shanghai, China; 2 Department of General Surgery, Shanghai Ninth People’s Hospital, Shanghai JiaoTong University School of Medicine, Shanghai, China; 3 Department of Forensic Sciences, Soochow University, Suzhou, China; 4 Department of Obstetrics and Gynecology, The Affiliated Suzhou Hospital of Nanjing Medical University, 242, Guangji Road, Suzhou, 215000 China

**Retraction Note: BMC Cancer 23, 96** **(2023)**

**https://doi.org/10.1186/s12885-023-10573-3**

The Editors have retracted this article. After publication, concerns were raised about some of the data in this Article. Specifically, similarities were detected between panels 2 and 3 showing migration in SKOV3 cells in Fig. 3E.

The Authors were unable to provide a sufficient response to concerns. Furthermore, the ethics approval documents provided by the authors appear to be for another study and the IHC staining of the mice tumors appears to pre-date the ethics approval document from the animal ethics committee. The Editors have therefore lost confidence in the data and conclusions of this article.

Authors Hongjuan Chai, Chunpeng Pan, Mingyang Zhang, Haizhong Huo, Haiyan Shan, and Jugang Wu did not respond to correspondence from the Publisher about this retraction.

